# A microRNA biomarker panel for the non-invasive detection of bladder cancer

**DOI:** 10.18632/oncotarget.13382

**Published:** 2016-11-16

**Authors:** Virginia Urquidi, Mandy Netherton, Evan Gomes-Giacoia, Daniel J. Serie, Jeanette Eckel-Passow, Charles J. Rosser, Steve Goodison

**Affiliations:** ^1^ Nonagen Bioscience Corporation, Jacksonville, FL, USA; ^2^ Cancer Research Institute, MD Anderson Cancer Center, Orlando, FL, USA; ^3^ Department of Health Sciences Research, Mayo Clinic, Jacksonville, FL USA; ^4^ Department of Health Sciences Research, Mayo Clinic, Rochester, MN USA; ^5^ University of Hawaii Cancer Center, Honolulu, HI USA; ^6^ Department of Urology, Mayo Clinic, Jacksonville, FL USA

**Keywords:** diagnostic biomarkers, bladder cancer, microRNA, multiplex, urinalysis

## Abstract

The development of accurate, non-invasive urinary assays for bladder cancer would greatly facilitate the detection and management of a disease that has a high rate of recurrence and progression. In this study, we employed a discovery and validation strategy to identify microRNA signatures that can perform as a non-invasive bladder cancer diagnostic assay. Expression profiling of 754 human microRNAs (TaqMan low density arrays) was performed on naturally voided urine samples from a cohort of 85 subjects of known bladder disease status (27 with active BCa). A panel of 46 microRNAs significantly associated with bladder cancer were subsequently monitored in an independent cohort of 121 subjects (61 with active BCa) using quantitative real-time PCR (RT-PCR). Multivariable modeling identified a 25-target diagnostic signature that predicted the presence of BCa with an estimated sensitivity of 87% at a specificity of 100% (AUC 0.982). With additional validation, the monitoring of a urinary microRNA biomarker panel could facilitate the non-invasive evaluation of patients under investigation for BCa.

## INTRODUCTION

The development of accurate assays that can detect and monitor bladder cancer (BCa) non-invasively through urinalysis would benefit both patients and healthcare systems. A number of tests have been developed to detect bladder tumor-associated urinary biomarkers, but due to inadequate sensitivity and poor overall accuracy, none of these assays have sufficient predictive power to be applied to the management of individual patients [1]. A shift from single biomarker assays [2] to multiplex molecular signatures that reflect the multiple pathways evident in BCa development provides an opportunity to develop assays with clinical utility for a breadth of diagnostic scenarios.

In previous studies, we demonstrated the feasibility of profiling the mRNA transcriptome of urothelia obtained from naturally micturated urine and developed an analytical approach to identify cancer-associated gene signatures. Genome-wide expression profiling and validation of selected candidate biomarkers in an independent cohort of subjects identified multiplex mRNA signatures that achieved promising diagnostic performance [3–5]. In this study, we extended this discovery and validation strategy to identify microRNA (miRNA) biomarkers for potential inclusion in a non-invasive BCa detection assay.

From the profiling of 754 human miRNAs in a cohort of 85 subjects, 46 miRNAs were identified as being significantly associated with the presence of BCa, and these were monitored in an independent validation cohort of 121 subjects using quantitative real-time PCR (RT-PCR). Multivariable modeling identified multiplex miRNA signatures that achieved encouraging diagnostic performance values. This phase II biomarker development study [6] confirms the potential of using urothelial cell RNA signatures for the non-invasive detection of BCa. The described miRNA biomarkers and predictive models warrant additional investigation for potential application in urology patient evaluation.

## RESULTS

Urine samples were collected consecutively from a total of 206 subjects, of which, 88 subjects had biopsy-proven, active BCa. Samples from the first 85 subjects collected (discovery-cohort 1) were profiled to identify candidate miRNA markers, and samples from the subsequent 121 subjects (validation-cohort 2) were used to assess the potential utility of the selected markers in an independent analysis. Demographic and clinicopathological details of cases and controls for each cohort are provided in Table 1. Gender distribution (3–4 times more men than women), and older patients in the BCa group reflect typical BCa incidence statistics [7].

**Table 1:** Demographic and clinicopathologic characteristics of study cohorts

	Cohort 1				Cohort 2			
	Controls *N* = 58		Cases *N* = 27		Controls *N* = 60		Cases *N* = 61	
**Median Age** (range, years)	61	(20–88)	66	(52–87)	60.5	(19–90)	70	(29–94)
**Gender**								
Male	47	(82.5%)	19	(70.4%)	47	(79.7%)	50	(86.2%)
Female	10	(17.5%)	7	(25.9%)	12	(20.3%)	8	(13.8%)
Missing	1		1		1		3	
**Race**								
White	38	(65.5%)	23	(85.2%)	42	(70.0%)	44	(72.1%)
African American	7	(12.1%)	1	(3.7%)	3	(3.0%)	5	(8.2%)
Other	4	(6.90%)	1	(3.7%)	4	(6.7%)	5	(8.2%)
Unknown	9		2		11		7	
**Clinical stage**								
Ta	n/a		4	(14.8%)	n/a		15	(27.8%)
Cis	n/a		3	(11.1%)	n/a		6	(11.1%)
T1	n/a		9	(33.3%)	n/a		13	(24.1%)
T2	n/a		7	(25.9%)	n/a		15	(27.8%)
T3	n/a		3	(11.1%)	n/a		5	(9.3%)
Missing			1				7	
**Grade**								
High	n/a		22	(81.5%)	n/a		41	(83.7%)
Low	n/a		1	(3.7%)	n/a		8	(16.3%)
Missing	n/a		4		n/a		12	
**Hematuria**								
No	51	(94.4%)	19	(70.4%)	49	(90.7%)	45	(77.6%)
Yes	3	(5.6%)	6	(22.2%)	5	(9.3%)	13	(22.4%)
Missing	4		2		6		3	
**Cytology results**								
Negative	n/a		11	(40.7%)	n/a		20	(42.6%)
Positive	n/a		8	(29.6%)	n/a		22	(46.8%)
Reactive	n/a		2	(7.4%)	n/a		3	(6.4%)
Suspicious	n/a		2	(7.4%)	n/a		2	(4.3%)
Missing			4		0		14	

### Urothelial cell miRNA profiling

A panel of 754 human miRNAs was monitored in a set of urothelial samples obtained from a total of 85 subjects of known bladder disease status, 27 of which had biopsy-proven BCa (cohort 1). Of the 754 miRNAs included on the Taqman low density arrays (Human MicroRNA Set v3.0), 267 were detected in at least one urothelial cell sample. Comparative group (cases vs. controls) analysis identified 108 miRNAs that were significantly associated (*P* < 0.05) with BCa status. A set of 46 miRNAs (Supplementary Table S1) from the broad-spectrum, discovery profiling was selected by statistical ranking and absolute fold-change (> 2.0) between cases and controls for validation in an independent cohort.

### Association of candidate miRNA biomarkers with bladder cancer

The 46 candidate miRNA biomarkers were tested in urine samples obtained from an independent cohort comprised of 121 subjects, 61 with confirmed BCa (Table 1). RT-PCR analysis confirmed that the control miRNA was detected in all samples tested. Expression of the candidate miRNA markers across samples is presented in Table 2. To avoid bias introduced by the issue of RT-PCR non-detects [8] we employed a left-censoring statistical approach to determine per-target differential expression in cases versus controls. Table 2 provides univariate differential expression results for each biomarker, ranked by Tobit model *P*-value [9]. Additional information on biomarker candidacy was obtained by evaluating the association with specific clinical factors or distinct subsets of patients. Left-censored Tobit models were used to estimate and compare associations of biomarkers with clinical factors (hematuria, tumor grade, clinical stage, age, sex). Very few of the top-ranked candidate biomarkers (Tobit model *P* < 0.05) were significantly associated with gender (Supplementary Table S2), age or hematuria (four, three and zero, respectively). Four miRNAs were significantly associated with tumor grade, and three with muscle-invasive disease. Notably, miR-199a-3p was associated with grade, invasive disease, age, and sex. Although no targets were excluded for the analyses presented here, such identified associations could impact decisions regarding inclusion in future tests for a specific clinical utility.

**Table 2:** Univariate Tobit model results for testing the association of 46 candidate miRNA biomarkers with case-control status

		% Samples Censored		Association with BCa	
miRNA	AB Assay ID	Controls	Cases	Estimate	*P*-value
hsa-miR-140-5p	4373374	0.25	0.05	2.11	6.50E–06
hsa-miR-142-5p	4395359	0.44	0.11	2.91	7.85E–06
hsa-miR-199a-3p	4395415	0.56	0.18	3.18	8.49E–06
hsa-miR-93	4373302	0.22	0.02	2.00	9.23E–06
hsa-miR-652	4395463	0.30	0.07	2.37	1.82E–05
hsa-miR-20a	4373286	0.10	0.02	1.46	4.17E–04
hsa-miR-106b*	2380	0.29	0.10	1.83	4.50E–04
has-miR-1305	2867	0.27	0.53	−7.48	4.60E–04
hsa-miR-223	4395406	0.05	0.00	1.64	6.19E–04
hsa-miR-18a	4395533	0.42	0.11	2.00	7.30E–04
hsa-miR-191	4395410	0.06	0.00	0.86	9.56E–04
hsa-miR-126	4395339	0.16	0.01	1.93	1.42E–03
hsa-miR-26b	4395167	0.14	0.02	1.33	2.71E–03
hsa-miR-26a	4395166	0.17	0.03	1.26	5.23E–03
hsa-miR-145	4395389	0.36	0.10	1.54	5.75E–03
hsa-miR-146a	4373132	0.14	0.00	1.38	6.43E–03
hsa-miR-30a-3p	416	0.03	0.01	−1.81	6.60E–03
hsa-miR-96	4373372	0.64	0.34	2.67	9.62E–03
hsa-miR-573	1615	0.45	0.40	6.91	1.14E–02
hsa-miR-221	4373077	0.40	0.18	2.30	1.49E–02
hsa-miR-182	4395445	0.36	0.07	1.27	1.83E–02
hsa-miR-142-3p	4373136	0.09	0.00	0.89	4.03E–02
hsa-miR-19b	4373098	0.06	0.02	0.79	4.84E–02
hsa-miR-224	4395210	0.30	0.07	1.24	5.16E–02
hsa-miR-181a	4373117	0.22	0.06	0.67	7.47E–02
hsa-miR-766	1986	0.27	0.26	−1.17	1.09E–01
hsa-miR-146b-5p	4373178	0.23	0.03	0.74	1.10E–01
hsa-miR-429	4373203	0.16	0.03	0.91	1.20E–01
hsa-miR-200a	4378069	0.28	0.07	0.83	1.42E–01
hsa-miR-200c	4395411	0.08	0.02	0.83	1.44E–01
hsa-miR-20b	4373263	0.29	0.08	0.80	1.50E–01
hsa-miR-324-3p	4395272	0.25	0.07	0.72	1.50E–01
hsa-miR-19a	4373099	0.22	0.06	0.63	1.58E–01
hsa-miR-106a	4395280	0.10	0.05	0.70	1.69E–01
hsa-miR-143	4395360	0.55	0.31	1.16	2.01E–01
hsa-miR-99b	4373007	0.28	0.05	0.56	2.20E–01
hsa-miR-140-3p	4395345	0.26	0.10	0.56	2.40E–01
hsa-miR-491-5p	4381053	0.28	0.11	0.55	2.56E–01
hsa-miR-151-3p	2254	0.04	0.01	0.48	3.04E–01
hsa-miR-671-3	4395433	0.57	0.39	−1.08	3.04E-01
hsa-miR-222	4395387	0.07	0.00	0.29	4.68E-01
hsa-miR-339-3p	4395295	0.27	0.07	0.24	6.17E-01
hsa-miR-141	4373137	0.18	0.03	−0.18	7.17E-01
hsa-miR-200b	4395362	0.31	0.14	−0.27	7.31E-01
hsa-let-7b	4395446	0.23	0.07	−0.17	7.56E-01
hsa-miR-21	4373090	0.10	0.03	−0.09	8.19E-01

AB, Applied Biosystems. BCa, bladder cancer

Biomarkers are ranked by Tobit model *P*-value. Because of censoring, the Tobit model estimate represents the difference between cases and controls in the un-observed latent variable. The percent of cases and controls censored is provided.

### Multivariable analysis and prediction modeling

**Figure 1:**
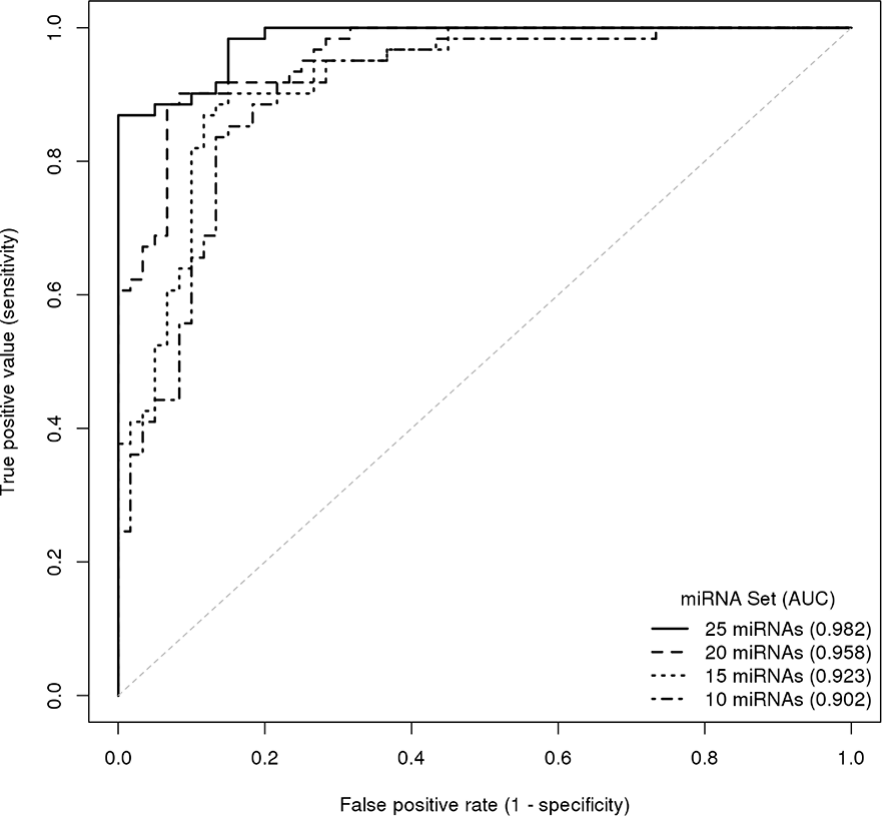
ROC curve illustrating the diagnostic accuracy of miRNA set classifiers for predicting the presence of bladder cancer Curves are presented for prediction models comprised of 25, 20, 15 and 10 miRNAs.

Multivariable logistic models were constructed from the 121-cohort data to identify a multivariable model that could predict the case-control status of a given sample. The LASSO approach [10] was used to shrink model coefficients, and model performance was described using receiver operating characteristic (ROC) analysis [11]. Corresponding odds ratios from the multivariable logistic regression models are shown in Table 3. This analysis identified a 25-miRNA prediction model (see Supplementary Table S3) with estimates of 87% sensitivity and 100% specificity (AUC 0.982). The restriction of candidate biomarkers included in the modeling resulted in a reduction of overall model performance but also shifted the balance of sensitivity and specificity estimates (Figure 1). A 20-miRNA prediction model (AUC 0.958) had an improved sensitivity (93%) and reduced specificity (88%). Even a 10-miRNA prediction model achieved good overall performance (AUC 0.902) with a sensitivity of 84% and specificity of 87% (Figure 1). Of the 59 cases in this cohort that had VUC data available, VUC evaluation positively identified 47%. The comparison across prediction models revealed which miRNAs contribute to performance (Table 1) and can aid in the selection of optimal assays for future clinical development.

**Table 3:** Multivariable logistic diagnostic models

miRNA	25-miRNA model	20-miRNA model	15-miRNA model	10-miRNA model
hsa-miR-652	1.137	1.088	1.061	1.065
hsa-miR-199a-3p	1.313	1.255	1.186	1.146
hsa-miR-140-5p	1.093	1.107	1.129	1.092
hsa-miR-93	1.462	1.243	1.113	1.119
hsa-miR-142-5p	1.048	1.043	1.043	1.031
has-miR-1305	0.807	0.862	0.894	0.921
hsa-miR-30a	0.907	0.880	0.870	0.946
hsa-miR-224	1.203	1.114	1.054	1.008
hsa-miR-96	1.109	1.084	1.048	1.020
hsa-miR-766	0.755	0.794	0.825	0.865
hsa-miR-223	1.135	1.037	1.024	
hsa-miR-99b	1.468	1.278	1.150	
hsa-miR-140-3p	0.881	0.935	0.990	
hsa-let-7b	0.556	0.774	0.968	
hsa-miR-141	0.677	0.833	0.998	
hsa-miR-191	1.694	1.129		
hsa-miR-146b-5p	0.854	0.988		
hsa-miR-491-5p	0.860	0.994		
hsa-miR-339-3p	0.892	0.987		
hsa-miR-200c	1.497	1.215		
hsa-miR-106b*	1.054			
hsa-miR-143	0.973			
hsa-miR-429	1.120			
hsa-miR-222	0.999			
hsa-miR-200a	1.003			

The Lasso method was used to shrink model coefficients. The corresponding odds ratios are provided for models comprised of 25, 20, 15 and 10 miRNAs.

## DISCUSSION

With an estimated 75,000 new cases each year in the US [7, 12], bladder cancer is a major cause of morbidity and mortality. Although not typically life-threatening if detected early, more than 70% of patients with BCa will have a recurrence during the first two years after diagnosis. This recurrence phenomenon means patients face a lifetime of surveillance undergoing multiple invasive procedures. Current guidelines support a diagnostic approach of cystoscopy coupled with voided urine cytology (VUC). Invasive cystoscopy is associated with significant discomfort, possible infection and trauma. VUC is a non-invasive adjunct to cystoscopy, but the assay has poor sensitivity, especially for low-grade and low-stage tumors [13]. A number of tests have been developed to detect tumor-associated urinary biomarkers, but due to poor sensitivity and overall accuracy, none of these assays have sufficient predictive power to be applied to the management of individual patients [6].

In this study, we were able to identify and validate a panel of miRNA biomarkers that can accurately detect bladder cancer using a non-invasive urinary assay. MiRNAs are a class of short, non-coding RNA molecules that modulate protein expression through the perturbation of mRNA translation. Complementary binding of miRNAs to target mRNA transcripts causes suppression of translation through interference of complex formation or mRNA degradation [14]. Each miRNA can have multiple targets so changes in the profile of expressed miRNAs can have magnified effects on cellular phenotype. Although far from fully characterized, specific microRNAs have been implicated in a number of diseases, including cancers [15, 16], and miRNA signatures are potential biomarkers of disease status.

In order to identify miRNA signatures with potential for non-invasive diagnosis, we employed a discovery and validation strategy using urothelial cell samples that are naturally shed from the bladder lining and can be readily recovered from urine [3, 4]. The rationale for analyzing the shed urothelial component of urine is two-fold. Firstly, the analysis of the component that will be the analyte of a future assay is optimal. Secondly, the analyte enables comparison of samples collected from subjects with non-malignant conditions. Conversely, truly normal bladder tissues are rarely available from surgically excised material.

The profiling of 754 miRNAs on commercially available arrays in one set of samples enabled the selection of 46 BCa-associated targets for quantitative validation in an independent cohort using a custom-designed TLDA. The validation analysis included the application of a more stringent statistical approach. We used a left-censoring approach to handle RT-PCR non-detects [8]; reactions that fail to produce a signal above an arbitrarily pre-specified minimum. These non-detects are typically treated as ‘missing’ data leading to biased inference, so it is beneficial to use approaches that can reduce such bias when validating candidate gene expression biomarkers. We subsequently used Tobit modeling to compare gene expression differences, because it is designed to estimate relationships between variables when there is censoring in the dependent variable [9].

Analysis of the validation cohort confirmed that the majority of the candidate miRNA biomarkers were associated with the presence of BCa, but association with specific clinical variables was much less evident. While association with age, gender or the presence of hematuria could negatively influence the inclusion of a biomarker for a desired broad spectrum BCa test, the inclusion of markers that are significantly associated with BCa *plus* stage or grade might provide additional information with regard to patient evaluation and management.

In multivariable analyses, a 25-miRNA model was able to predict the presence of bladder disease with high sensitivity (87%) while maintaining optimal specificity (100%). These values compare very favorably with those of cystoscopy and VUC, which both rely on high specificity for overall accuracy. Restriction to reduced target prediction models resulted in an expected reduction in performance values, but even a 10-miRNA model achieved sensitivity of 84% and specificity of 87% (AUC 0.902). The model performance values also compare well with existing urinary tests for BCa detection. To date, there are four urine tests that have received FDA approval for diagnostic clinical use (BTA-Stat, BTA-Trak, NMP22 POC device, UroVysion FISH test), and a couple of others with approval restricted to post-treatment monitoring [6]. In a meta-analysis of 57 studies [17], although specificity of the current diagnostic tests was in the range of 74% to 88%, none achieved a sensitivity > 69%. The limiting factor for these tests may be the reliance on single biomarkers or the inclusion of chromosomal changes that are known to be restricted to a subset of BCa patients.

A number of studies have investigated the expressed miRNA repertoire in excised bladder tumor tissues and identified specific miRNAs that inform tumor stratification, predict outcome [18–21] or indicate the perturbation of specific molecular pathways [22–24]. More recently, a number of reports have described the potential utility of urinary miRNA monitoring for the purposes of BCa diagnosis. Hanke *et al*. reported that the ratio of miR-126:miR-152 in urine was indicative of BCa [25], Miah *et al*. monitored 15 miRNAs in a cohort of 121 subjects [26] and identified a 3-miRNA panel that detected BCa with high sensitivity (94%) but poor specificity (51%), and Wang *et al*. described levels of the miR-200 family plus miR-192 and miR-155 associated with Bca [27], but the potential of these biomarkers has not been evaluated in validation studies.

As miRNA characterization and reagent availability has improved, a number of more comprehensive urinary profiling studies have been performed. In a study conducted in Spain, Mengual *et al*. monitored over 750 miRNAs in urine samples obtained from 40 subjects, and identified a 6-miRNA diagnostic biomarker panel that achieved 85% sensitivity and 86% specificity for BCa detection in an independent cohort [28]. In a US cohort, De Long *et al*. profiled 730 miRNAs in pooled samples from 130 subjects to identify a 4-miRNA panel that had a diagnostic sensitivity of 88% and specificity of 78%, but the panel was not tested in an independent cohort [29]. Most recently, an Australasian group [30] evaluated the potential utility of a 12-miRNA panel obtained from a literature search for the detection of BCa recurrence in 81 subjects. A panel comprised of 6 of the 12 miRNAs achieved sensitivity of 88% and specificity of 48% for the detection of recurrent BCa in an independent cohort of 50 subjects.

In our study, the optimal miRNA signature was comprised of 32 targets, and with improving quantitative PCR technologies the monitoring of multiplex RNA panels is not a limiting factor. The limiting of prediction models to 15 and 10 miRNAs expectedly resulted in some loss of performance, but it also revealed several miRNAs (miR-140-5p, miR-199a-3p, miR-93, miR-652, miR-1305, miR-224, miR-96, miR-766) that consistently contributed to all models. Given the relatively small number of characterized miRNAs [31], there is inevitably some overlap in the miRNAs included in our panels with those in previous bladder cancer studies, but the majority of these core miRNAs have not been reported to be associated with BCa previously.

We recognize that the study has a number of limitations. Although both cases and controls were collected consecutively, molecular analyses were only initiated when balance of samples in each group was achieved. Firstly, disease prevalence is typically considerably lower in urologic practice, so evaluation of the validation study cohort is likely to provide an overly optimistic assessment of the assay predictive value. Secondly, as samples were collected prior to clinical evaluation for bladder cancer, other neoplastic urological conditions (kidney cancer, prostate cancer, benign prostatic hyperplasia) are under-represented in our control study cohort. It is also understood that biomarker performance values derived from limited validation studies can over-estimate potential value with respect to utility in larger, more diverse independent cohorts. Continued recruitment of patients in ongoing in order to perform larger studies that will better represent urological disease prevalence, and evaluate potential confounding comorbidities. As we expand the cohorts to include the breadth of urology clinic visitors, we will also endeavor to derive predictive models that might perform well for specific clinical scenarios, including first-event diagnosis or disease recurrence monitoring. If a highly sensitive microRNA test can be established, there is potential to reduce unnecessary, invasive cystoscopy procedures. Furthermore, beyond VUC, it will be of interest to directly compare the performance of a miRNA-based diagnostic signature with existing urine tests in the same samples. While there are issues with the measurement of labile RNA analytes, a number of RNA-based assays are being translated into tests that meet clinical laboratory standards [32].

The development of accurate assays that can detect and monitor bladder cancer non-invasively through urinalysis would be a major advance, benefiting both patients and healthcare systems, but this remains a challenge. In this study, we have identified urinary miRNA signatures that achieve encouraging diagnostic performance values. Additional, prospective studies are underway that will incorporate enough samples to stringently test the robustness of biomarkers and models [11, 15] and to evaluate the potential added value of the multiplex miRNA assay in clinical decision making.

## MATERIALS AND METHODS

### Clinical sampling and processing

Under IRB approval and informed consent, urine samples and associated clinical information were consecutively collected from subjects visiting the urology clinic at MD Anderson Cancer Center at Orlando, FL between 2010 and 2013. The discovery cohort consisted of 58 individuals with no evidence of active urothelial cell carcinoma (controls) and 27 individuals with primary urothelial carcinoma (cases). The validation cohort consisted of 60 individuals with no evidence of active urothelial cell carcinoma (controls) and 61 individuals with newly diagnosed primary urothelial carcinoma (cases). All subjects underwent standard clinical work-up, including office cystoscopy, and the majority also had axial imaging of the abdomen and pelvis. For the bladder cancer case group, histological confirmation of urothelial carcinoma, including grade and stage was defined from excised tissue. A summary of clinical data for both cohorts is given in Table 1. Prior to any intrusive investigation or treatment, 30–50 ml of midstream voided urine was collected from each subject in a sterile cup and stored at 4°C until processing (< 3 hrs.). Each sample was assigned a unique identifying number before laboratory processing. Urothelial cells were pelleted from the total urine sample by centrifugation (600 × g, 4°C, 5 min), rinsed in PBS, pelleted again, and frozen for storage at −80°C. Total RNA was purified using Qiagen RNeasy kit with subsequent Qiagen DNase treatment. RNA samples were evaluated quantitatively (at least 25 ng per sample obtained) and qualitatively using an Agilent Bioanalyzer 2000, before storage at −80°C as previously described [3, 4].

### Quantitative real-time PCR analysis

Profiling of 754 human miRNAs was performed using TaqMan^®^ Array Human MicroRNA A+B Cards Set v3.0 (Applied Biosystems Cat# 4444913). The Taqman low density array (TLDA) format is a 384-well system that uses standard TaqMan assays and enables automated loading and high-throughput analyses [33]. Details on the included assays are available in the Target List file (Applied Biosystems website), and each array included an endogenous control (Mamm U6) for data normalization. Megaplex™ RT Primers, Human Pool Set v3.0 and Megaplex™ PreAmp Primers (Applied Biosystems) were used for cDNA synthesis and preamplification respectively. Custom TLDAs for the validation studies were constructed by Applied Biosystems (AB) upon request. Targets included Mamm U6 as endogenous control plus 46 miRNAs biomarkers identified as associated with the presence of BCa from the discovery profiling analysis. Targets for validation were selected by statistical ranking (*P*-value) and fold-change, including 4 targets negatively associated with BCa. The complete list of targets selected for validation and Applied Biosystems TaqMan assay ID details are listed in Supplementary Table S1. The PCR reactions were run on a 7900HT Fast Real-Time PCR System (Applied Biosystems). RT-PCR amplification results were processed with RQ manager (Applied Biosystems). The baseline correction was manually checked for each target and the Ct threshold was set to 0.2 for every target across all plates. Targets deemed to be undetermined (Ct > 40) were given a Ct 40 value.

### Statistical analysis

For the 754-target profiling analyses, Delta Ct (DCt) values from duplicate data were calculated by normalization with the endogenous reference Mamm U6 miRNA and the fold-change between bladder cancer and control samples was calculated as log 2 ^-DDCT^. Differential miRNA expression between bladder cancer cases and controls was determined using Student's t-statistic. For the validation study analyses (cohort 2), differences in clinical covariates between bladder cancer cases and non-malignant controls were evaluated via Chi-squared test and Wilcoxon Rank Sum test, as appropriate. For each miRNA, the percentage of samples that were censored (Ct value = 40) was calculated for cases and controls separately (Supplementary Table S1). To avoid biased inference caused by the issue of RT-PCR non-detects (Ct value = 40), we used a left-censoring approach. Ct values of 40 were substituted with the highest observed Ct value for a given miRNA [8]. Ct values were then normalized by subtracting the Ct value of the endogenous control (Mamm U6) from each of the 46 miRNAs of interest. For each miRNA, left-censored Tobit models [9] were used to test for differences in miRNA expression between cases and controls. Multivariable logistic models were employed on the 121-cohort data to develop a signature to predict bladder cancer diagnosis. All microRNAs with <50% censoring were considered in the multivariable models and lasso was used to shrink the model coefficients; shrinkage coefficients were determined from 10-fold cross validation to yield models comprised of 25, 20, 15 and 10 miRNA targets. As the number of miRNAs included in multivariable models increased above 25, predictive ability continued to improve but the model coefficients became increasingly unstable. ROC curves and associated AUCs were calculated to assess the performance of the multivariable models. The sensitivity and specificity associated with the maximum Youden index [34] was selected from each ROC curve. Left-censored Tobit models [9] were additionally used to evaluate associations between miRNA expression and clinical variables. Results with *P* < 0.05 were deemed statistically significant.

## SUPPLEMENTARY MATERIALS TABLES






