# Supplementary material for: A microRNA biomarker panel for the non-invasive detection of bladder cancer
Source: Oncotarget. 2016 Nov 16;7(52):86290–9. doi: 10.18632/oncotarget.13382 (PMC5349914; doi:10.18632/oncotarget.13382)
Supplement: Supplementary file 1 [file oncotarget-07-86290-s001.pdf]

A microRNA biomarker panel for the non-invasive detection of bladder cancer

Supplementary Materials

Supplementary Table S1: miRNA Profiling RT-PCR Data. See Supplementary_Table_S1

Supplementary Table S2: Association of miRNA biomarkers with clinical variables. See Supplementary_Table_S2
