# Supplementary material for: A microRNA biomarker panel for the non-invasive detection of bladder cancer
Source: Oncotarget. 2016 Nov 16;7(52):86290–9. doi: 10.18632/oncotarget.13382 (PMC5349914; doi:10.18632/oncotarget.13382)
Supplement: Supplementary file 2 [file oncotarget-07-86290-s002.docx]

**Supplemental Table 1.** miRNA profiling RT-PCR data. Profiling (754 targets) was performed on an 85-subject cohort (cohort 1). Differential expression data for 46 miRNA targets selected for validation (Table 2) are shown.

| **miRNA** | **DCt Cases (mean)** | | **DCt Controls (mean)** | | **DDCt** | | **Fold**  **Change** | | **t-test  *P* value** | |  |
| --- | --- | --- | --- | --- | --- | --- | --- | --- | --- | --- | --- |
| hsa-miR-140-5p | 0.69 | 4.86 | | -4.17 | | 18.06 | | <0.0001 | |  |  |
| hsa-miR-142-5p | 6.46 | 9.50 | | -3.04 | | 8.25 | | <0.0001 | |  |  |
| hsa-miR-199a-3p | 6.80 | 10.62 | | -3.82 | | 14.11 | | <0.0001 | |  |  |
| hsa-miR-93 | -0.78 | 3.42 | | -4.20 | | 18.33 | | <0.0001 | |  |  |
| hsa-miR-652 | 3.66 | 7.50 | | -3.84 | | 14.30 | | <0.0001 | |  |  |
| hsa-miR-20a | -0.19 | 2.29 | | -2.48 | | 5.58 | | <0.0001 | |  |  |
| hsa-miR-106b* | 6.60 | 9.47 | | -2.86 | | 7.28 | | <0.0001 | |  |  |
| has-miR-1305 | 9.19 | 2.43 | | 6.75 | | -108.00 | | <0.0001 | |  |  |
| hsa-miR-223 | -7.59 | -4.18 | | -3.40 | | 10.58 | | <0.0001 | |  |  |
| hsa-miR-18a | 5.76 | 8.84 | | -3.08 | | 8.48 | | <0.0001 | |  |  |
| hsa-miR-191 | -3.35 | -0.71 | | -2.64 | | 6.24 | | <0.0001 | |  |  |
| hsa-miR-126 | 2.63 | 5.73 | | -3.09 | | 8.52 | | <0.0001 | |  |  |
| hsa-miR-26b | 0.34 | 2.67 | | -2.33 | | 5.02 | | <0.0001 | |  |  |
| hsa-miR-26a | -0.50 | 2.43 | | -2.93 | | 7.61 | | <0.0001 | |  |  |
| hsa-miR-145 | 2.92 | 7.76 | | -4.85 | | 28.74 | | <0.0001 | |  |  |
| hsa-miR-146a | -0.28 | 2.47 | | -2.75 | | 6.71 | | <0.0001 | |  |  |
| hsa-miR-30a-3p | 2.76 | 1.27 | | 1.48 | | -2.80 | | 0.0014 | |  |  |
| hsa-miR-96 | 10.21 | 11.61 | | -1.40 | | 2.63 | | 0.0166 | |  |  |
| hsa-miR-573 | 8.48 | 5.26 | | 3.22 | | -9.31 | | 0.0052 | |  |  |
| hsa-miR-221 | 4.91 | 7.20 | | -2.29 | | 4.89 | | 0.0003 | |  |  |
| hsa-miR-182 | 5.45 | 8.49 | | -3.04 | | 8.25 | | <0.0001 | |  |  |
| hsa-miR-142-3p | -1.05 | 1.76 | | -2.81 | | 7.00 | | <0.0001 | |  |  |
| hsa-miR-19b | -1.81 | 0.67 | | -2.48 | | 5.58 | | <0.0001 | |  |  |
| hsa-miR-224 | 3.49 | 5.81 | | -2.31 | | 4.97 | | 0.0008 | |  |  |
| hsa-miR-181a | 5.53 | 7.61 | | -2.07 | | 4.21 | | <0.0001 | |  |  |
| hsa-miR-766 | 8.51 | 9.71 | | -1.20 | | 2.30 | | 0.0282 | |  |  |
| hsa-miR-146b-5p | 1.10 | 4.57 | | -3.48 | | 11.14 | | <0.0001 | |  |  |
| hsa-miR-429 | 2.56 | 4.45 | | -1.89 | | 3.70 | | 0.0005 | |  |  |
| hsa-miR-200a | 1.89 | 4.97 | | -3.08 | | 8.46 | | <0.0001 | |  |  |
| hsa-miR-200c | -2.15 | -0.28 | | -1.87 | | 3.65 | | 0.0025 | |  |  |
| hsa-miR-20b | 2.95 | 6.17 | | -3.22 | | 9.32 | | <0.0001 | |  |  |
| hsa-miR-324-3p | 5.01 | 7.21 | | -2.19 | | 4.57 | | <0.0001 | |  |  |
| hsa-miR-19a | 3.04 | 5.90 | | -2.85 | | 7.23 | | <0.0001 | |  |  |
| hsa-miR-106a | -0.79 | 1.43 | | -2.21 | | 4.64 | | 0.0002 | |  |  |
| hsa-miR-143 | 7.81 | 10.49 | | -2.68 | | 6.41 | | 0.0003 | |  |  |
| hsa-miR-99b | 4.28 | 7.58 | | -3.30 | | 9.82 | | <0.0001 | |  |  |
| hsa-miR-140-3p | 6.59 | 8.76 | | -2.17 | | 4.50 | | <0.0001 | |  |  |
| hsa-miR-491-5p | 5.77 | 7.79 | | -2.01 | | 4.03 | | 0.0002 | |  |  |
| hsa-miR-151-3p | -0.58 | -2.34 | | 1.76 | | -3.39 | | 0.0159 | |  |  |
| hsa-miR-671-3p | 9.87 | 11.03 | | -1.17 | | 2.24 | | 0.0455 | |  |  |
| hsa-miR-222 | -3.00 | -1.46 | | -1.54 | | 2.91 | | 0.0054 | |  |  |
| hsa-miR-339-3p | 6.06 | 7.99 | | -1.93 | | 3.81 | | 0.0002 | |  |  |
| hsa-miR-141 | 3.41 | 4.77 | | -1.36 | | 2.56 | | 0.0127 | |  |  |
| hsa-miR-200b | 1.63 | 4.43 | | -2.80 | | 6.94 | | 0.0031 | |  |  |
| hsa-let-7b | -0.50 | 2.58 | | -3.08 | | 8.43 | | 0.0006 | |  |  |
| hsa-miR-21 | -0.61 | 0.44 | | -1.05 | | 2.07 | | 0.0475 | |  |  |
| DDCt= DCt (cases) – DCt (controls)  Fold Change (cases/controls) = 2 ^-DDCT^  Negative FC values indicate target is down-regulated in bladder cancer cases | | | | | | | | | | | |
