# Supplementary material for: A microRNA biomarker panel for the non-invasive detection of bladder cancer
Source: Oncotarget. 2016 Nov 16;7(52):86290–9. doi: 10.18632/oncotarget.13382 (PMC5349914; doi:10.18632/oncotarget.13382)
Supplement: Supplementary file 3 [file oncotarget-07-86290-s003.docx]

**Supplemental Table 2**. Association of miRNA biomarkers with clinical variables. Biomarkers are ranked by Tobit model estimate *P*-value (difference in mean expression between bladder cancer cases and controls). Hematuria; association with gross hematuria. Grade; association with high-grade versus low grade. Muscle-invasive disease; stage information grouped (non-invasive stages Tis, Ta, T1 versus invasive stages T2, T3).

|  |  | **Bladder Cancer** | | **Hematuria** | | **Grade** | | **Muscle Invasive** | | **Age** | | **Sex** | |
| --- | --- | --- | --- | --- | --- | --- | --- | --- | --- | --- | --- | --- | --- |
| **miRNA** | **AB  Assay ID** | **Estimate** | **P-value** | **Estimate** | **P-value** | **Estimate** | **P-value** | **Estimate** | **P-value** | **Estimate** | **P-value** | **Estimate** | **P-value** |
| hsa-miR-140-5p | 4373374 | 2.11 | 6.50E-06 | -0.775 | 4.65E-01 | 1.850 | 1.72E-01 | 0.553 | 5.81E-01 | 0.020 | 6.47E-01 | 0.374 | 7.68E-01 |
| hsa-miR-142-5p | 4395359 | 2.91 | 7.85E-06 | -0.902 | 3.83E-01 | 1.782 | 1.48E-01 | 1.607 | 7.56E-02 | -0.003 | 9.32E-01 | 0.321 | 7.80E-01 |
| hsa-miR-199a-3p | 4395415 | 3.18 | 8.49E-06 | 0.322 | 8.17E-01 | 4.571 | 8.01E-03 | 3.337 | 4.76E-03 | 0.108 | 4.06E-02 | 4.124 | 8.90E-03 |
| hsa-miR-93 | 4373302 | 2.00 | 9.23E-06 | 0.350 | 7.28E-01 | 0.971 | 4.26E-01 | 0.432 | 6.29E-01 | 0.030 | 4.27E-01 | 0.224 | 8.40E-01 |
| hsa-miR-652 | 4395463 | 2.37 | 1.82E-05 | 0.813 | 4.92E-01 | 1.831 | 1.88E-01 | 0.707 | 5.02E-01 | 0.000 | 9.94E-01 | 1.063 | 4.34E-01 |
| hsa-miR-20a | 4373286 | 1.46 | 4.17E-04 | 0.310 | 6.92E-01 | 0.763 | 4.13E-01 | 0.651 | 3.38E-01 | -0.020 | 4.96E-01 | -0.175 | 8.39E-01 |
| hsa-miR-106b | 2380 | 1.83 | 4.50E-04 | -0.252 | 7.90E-01 | 1.754 | 1.14E-01 | 1.501 | 6.69E-02 | 0.056 | 1.21E-01 | 1.058 | 3.04E-01 |
| hsa-miR-1305 | 2867 | -7.48 | 4.60E-04 | -0.779 | 8.97E-01 | -6.047 | 3.86E-01 | 3.116 | 5.70E-01 | -0.176 | 4.34E-01 | -8.132 | 2.11E-01 |
| hsa-miR-223 | 4395406 | 1.64 | 6.19E-04 | -1.149 | 1.29E-01 | 1.894 | 2.96E-02 | 1.202 | 7.27E-02 | 0.019 | 5.06E-01 | 0.410 | 6.31E-01 |
| hsa-miR-18a | 4395533 | 2.00 | 7.30E-04 | 0.314 | 7.77E-01 | 1.323 | 3.18E-01 | 1.286 | 1.87E-01 | 0.004 | 9.19E-01 | 0.013 | 9.92E-01 |
| hsa-miR-191 | 4395410 | 0.86 | 9.56E-04 | 0.064 | 9.19E-01 | 0.373 | 6.19E-01 | 0.112 | 8.40E-01 | 0.015 | 5.24E-01 | 0.608 | 3.76E-01 |
| hsa-miR-126 | 4395339 | 1.93 | 1.42E-03 | 1.510 | 1.12E-01 | 0.481 | 6.79E-01 | 0.701 | 4.10E-01 | 0.000 | 9.93E-01 | 0.280 | 7.93E-01 |
| hsa-miR-26b | 4395167 | 1.33 | 2.71E-03 | -0.311 | 6.71E-01 | 0.596 | 4.94E-01 | 0.314 | 6.27E-01 | -0.008 | 7.80E-01 | 0.048 | 9.53E-01 |
| hsa-miR-26a | 4395166 | 1.26 | 5.23E-03 | -0.609 | 4.74E-01 | 0.504 | 6.21E-01 | 0.028 | 9.70E-01 | 0.013 | 6.73E-01 | 0.310 | 7.40E-01 |
| hsa-miR-145 | 4395389 | 1.54 | 5.75E-03 | -1.107 | 3.86E-01 | 2.652 | 9.40E-02 | 1.755 | 1.27E-01 | 0.104 | 3.49E-02 | 0.995 | 5.01E-01 |
| hsa-miR-146a | 4373132 | 1.38 | 6.43E-03 | 0.716 | 3.60E-01 | 1.392 | 1.31E-01 | -0.150 | 8.27E-01 | 0.016 | 5.87E-01 | 1.242 | 1.49E-01 |
| hsa-miR-30a-3p | 416 | -1.81 | 6.60E-03 | 0.047 | 9.60E-01 | -1.018 | 3.45E-01 | -1.180 | 1.32E-01 | 0.005 | 8.76E-01 | 0.071 | 9.45E-01 |
| hsa-miR-96 | 4373372 | 2.67 | 9.62E-03 | -0.046 | 9.77E-01 | 3.085 | 1.22E-01 | 0.319 | 8.21E-01 | 0.080 | 1.90E-01 | -0.147 | 9.32E-01 |
| hsa-miR-573 | 1615 | 6.91 | 1.14E-02 | 0.199 | 9.54E-01 | -0.139 | 9.73E-01 | -0.044 | 9.88E-01 | -0.160 | 2.24E-01 | -3.029 | 4.22E-01 |
| hsa-miR-221 | 4373077 | 2.30 | 1.49E-02 | -0.002 | 9.99E-01 | 2.368 | 1.04E-01 | 2.350 | 2.95E-02 | 0.086 | 6.81E-02 | 0.179 | 8.96E-01 |
| hsa-miR-182 | 4395445 | 1.27 | 1.83E-02 | -0.294 | 8.09E-01 | 0.557 | 7.01E-01 | -0.692 | 5.13E-01 | 0.048 | 2.89E-01 | 0.657 | 6.23E-01 |
| hsa-miR-142-3p | 4373136 | 0.89 | 4.03E-02 | -1.175 | 9.73E-02 | 1.975 | 1.52E-02 | 0.946 | 1.33E-01 | 0.015 | 5.71E-01 | 0.508 | 5.25E-01 |
| hsa-miR-19b | 4373098 | 0.79 | 4.84E-02 | 0.266 | 7.66E-01 | 0.911 | 4.00E-01 | 0.678 | 3.90E-01 | 0.000 | 9.95E-01 | -0.317 | 7.47E-01 |
| hsa-miR-224 | 4395210 | 1.24 | 5.16E-02 | -0.571 | 6.04E-01 | 0.818 | 5.30E-01 | -0.120 | 9.00E-01 | 0.064 | 1.16E-01 | 2.435 | 3.98E-02 |
| hsa-miR-181a | 4373117 | 0.67 | 7.47E-02 | -0.019 | 9.81E-01 | 0.322 | 7.23E-01 | 0.314 | 6.37E-01 | 0.001 | 9.73E-01 | 1.537 | 6.65E-02 |
| hsa-miR-766 | 1986 | -1.17 | 1.09E-01 | -0.525 | 6.73E-01 | 0.486 | 7.33E-01 | 1.733 | 9.56E-02 | -0.018 | 6.99E-01 | -0.026 | 9.85E-01 |
| hsa-miR-146b-5p | 4373178 | 0.74 | 1.10E-01 | -0.205 | 8.30E-01 | 1.804 | 9.34E-02 | 0.678 | 3.96E-01 | 0.033 | 3.41E-01 | 1.464 | 1.53E-01 |
| hsa-miR-429 | 4373203 | 0.91 | 1.20E-01 | 0.511 | 5.74E-01 | 0.579 | 5.86E-01 | 0.108 | 8.91E-01 | 0.072 | 2.87E-02 | 0.826 | 4.07E-01 |
| hsa-miR-200a | 4378069 | 0.83 | 1.42E-01 | 0.500 | 6.71E-01 | 1.516 | 2.67E-01 | 0.382 | 7.03E-01 | 0.078 | 6.71E-02 | 2.655 | 3.55E-02 |
| hsa-miR-200c | 4395411 | 0.83 | 1.44E-01 | 0.538 | 5.98E-01 | 0.619 | 6.11E-01 | -0.160 | 8.58E-01 | 0.059 | 1.16E-01 | 0.777 | 4.88E-01 |
| hsa-miR-20b | 4373263 | 0.80 | 1.50E-01 | 1.171 | 2.99E-01 | 1.353 | 3.03E-01 | 0.705 | 4.61E-01 | 0.023 | 5.90E-01 | 0.218 | 8.62E-01 |
| hsa-miR-324-3p | 4395272 | 0.72 | 1.50E-01 | 0.997 | 2.12E-01 | 1.513 | 1.04E-01 | 0.810 | 2.35E-01 | 0.020 | 5.07E-01 | 0.553 | 5.33E-01 |
| hsa-miR-19a | 4373099 | 0.63 | 1.58E-01 | 0.657 | 4.86E-01 | 0.912 | 3.98E-01 | 0.355 | 6.53E-01 | 0.016 | 6.57E-01 | -0.537 | 6.06E-01 |
| hsa-miR-106a | 4395280 | 0.70 | 1.69E-01 | 0.835 | 3.82E-01 | 0.914 | 3.97E-01 | 0.967 | 2.18E-01 | 0.027 | 4.42E-01 | -0.535 | 6.11E-01 |
| hsa-miR-143 | 4395360 | 1.16 | 2.01E-01 | 0.052 | 9.79E-01 | 2.049 | 3.86E-01 | 3.244 | 5.60E-02 | 0.008 | 9.14E-01 | 3.455 | 1.33E-01 |
| hsa-miR-99b | 4373007 | 0.56 | 2.20E-01 | 0.059 | 9.48E-01 | 0.864 | 4.30E-01 | -0.450 | 5.72E-01 | 0.052 | 1.19E-01 | 1.285 | 1.98E-01 |
| hsa-miR-140-3p | 4395345 | 0.56 | 2.40E-01 | 0.231 | 8.02E-01 | 2.092 | 4.97E-02 | 1.408 | 7.59E-02 | 0.052 | 1.33E-01 | 1.106 | 2.77E-01 |
| hsa-miR-491-5p | 4381053 | 0.55 | 2.56E-01 | -0.604 | 4.91E-01 | 1.457 | 1.69E-01 | 0.961 | 2.26E-01 | 0.027 | 4.27E-01 | 1.620 | 9.51E-02 |
| hsa-miR-151-3p | 2254 | 0.48 | 3.04E-01 | 1.015 | 4.18E-01 | -2.231 | 1.32E-01 | -0.768 | 4.89E-01 | -0.057 | 2.33E-01 | -1.208 | 3.89E-01 |
| hsa-miR-671-3p | 4395433 | -1.08 | 3.04E-01 | 0.606 | 6.86E-01 | 1.102 | 5.44E-01 | 1.599 | 2.03E-01 | 0.042 | 4.66E-01 | 2.022 | 2.35E-01 |
| hsa-miR-222 | 4395387 | 0.29 | 4.68E-01 | 0.433 | 5.31E-01 | 0.253 | 7.59E-01 | -0.413 | 4.94E-01 | 0.007 | 7.90E-01 | 0.715 | 3.45E-01 |
| hsa-miR-339-3p | 4395295 | 0.24 | 6.17E-01 | 0.758 | 3.19E-01 | -0.328 | 7.14E-01 | 0.395 | 5.47E-01 | 0.019 | 4.96E-01 | 0.081 | 9.20E-01 |
| hsa-miR-141 | 4373137 | -0.18 | 7.17E-01 | 0.277 | 7.28E-01 | 0.335 | 7.20E-01 | 0.060 | 9.30E-01 | 0.040 | 1.69E-01 | 0.276 | 7.53E-01 |
| hsa-miR-200b | 4395362 | -0.27 | 7.31E-01 | 1.811 | 3.15E-01 | 1.089 | 6.17E-01 | 0.431 | 7.85E-01 | 0.116 | 7.90E-02 | 5.686 | 3.15E-03 |
| hsa-let-7b | 4395446 | -0.17 | 7.56E-01 | 0.669 | 6.23E-01 | 2.300 | 1.47E-01 | 0.009 | 9.93E-01 | 0.020 | 6.92E-01 | 2.702 | 6.57E-02 |
| hsa-miR-21 | 4373090 | -0.09 | 8.19E-01 | 0.189 | 7.67E-01 | 0.529 | 4.71E-01 | 0.289 | 5.89E-01 | 0.001 | 9.63E-01 | 0.320 | 6.51E-01 |
